# Detection of Corneal Ulcer Using a Genetic Algorithm-Based Image Selection and Residual Neural Network

**DOI:** 10.3390/bioengineering10060639

**Published:** 2023-05-24

**Authors:** Tugba Inneci, Hasan Badem

**Affiliations:** 1 Department of Informatics System, Kahramanmaras Sutcu Imam University, Kahramanmaras 46050, Türkiye; tugbainneci33@gmail.com; 2 Department of Computer Engineering, Kahramanmaras Sutcu Imam University, Kahramanmaras 46050, Türkiye

**Keywords:** corneal ulcer, deep neural network, feature maps, genetic algorithm, ResNet, transfer learning

## Abstract

Corneal ulcer is one of the most devastating eye diseases causing permanent damage. There exist limited soft techniques available for detecting this disease. In recent years, deep neural networks (DNN) have significantly solved numerous classification problems. However, many samples are needed to obtain reasonable classification performance using a DNN with a huge amount of layers and weights. Since collecting a data set with a large number of samples is usually a difficult and time-consuming process, very large-scale pre-trained DNNs, such as the AlexNet, the ResNet and the DenseNet, can be adapted to classify a dataset with a small number of samples, through the utility of transfer learning techniques. Although such pre-trained DNNs produce successful results in some cases, their classification performances can be low due to many parameters, weights and the emergence of redundancy features that repeat themselves in many layers in som cases. The proposed technique removes these unnecessary features by systematically selecting images in the layers using a genetic algorithm (GA). The proposed method has been tested on ResNet on a small-scale dataset which classifies corneal ulcers. According to the results, the proposed method significantly increased the classification performance compared to the classical approaches.

## 1. Introduction

Corneal ulcers are open sores in the eye’s cornea layer and affect the epithelial layer or the corneal stroma [1,2]. Corneal ulcers are the most frequently occurring symptom of corneal diseases due to contact lenses, trauma, adnexal diseases, topical steroid uses, severe debilitation, and ocular surface disorders [3]. The image of the eye stained with fluorescein is recorded by a camera mounted on the biomicroscope to determine the inflammatory wound’s position and severity [4]. How the cornea of the eye images is dyed (brightness, position and amount, etc.) is used to diagnose corneal ulcers in optometry and ophthalmology. The main solution is early diagnosis, which is a crucial step in preventing the effect of corneal ulcers [5]. However, the detection of corneal ulcers requires high-quality facilities and ophthalmologists, which are not available in developing countries. Therefore, efficient alternative machine learning techniques can be used to support the ophthalmologist to diagnose corneal ulcers [6,7].

The detection of the corneal ulcer from an image is three steps, including preprocessing, feature extraction, and classification. To attain efficient classification results, these three phases need to be planned properly [6,8,9]. In the first step, the noise level of the image is decreased, and the image segmentation is applied to separate the eye regions. After that, the features are extracted from the image. In the last step, features and their related labels are divided into two parts, including, training, and testing data set. The training data set is fed to a convenient classifier to tune the inner parameter of the classifier. Once the training is completed, the classifier is ready for the testing process [10].

However, the traditional machine learning techniques, such as k-nearest neighbors, support vector machine (SVM), decision tree, etc., have several disadvantages, including requiring several user-supplied parameters for the main three steps, sensitivity to outliers, overfitting, etc. [11,12,13,14]. In addition, choosing proper feature extraction and classification techniques is tedious and time-consuming [15,16]. The classification performance of the algorithm also decreases dramatically as the number of features and samples increases [17]. Recently, the DNN decreases these drawbacks, thanks to its capabilities, which are automatic feature extraction, and efficient classification results [18,19,20,21]. Moreover, feature selection between feature extraction and classification can be implemented to improve the success of the DNN [22]. However, the DNN requires too many training data samples and several convenient hyperparameters, including layer numbers, neuron numbers, optimization parameters, etc. [23]. Therefore, it is not possible to apply the DNN to solve small-scale data sets with a limited number of samples [23].

The transfer learning technique is applied to adapt the DNN for small-scale data sets [5,24,25], such as corneal data sets, with 720 samples being used in this study. Transfer learning provides the capability of DNN to extract features and the ability to use tuned hyperparameters [26]. The massive DNNs, including the AlexNet, the ResNet, the GoogleNet, the DenseNet, etc., have been trained with a large-scale data set called ImageNet [27] with 1 million images with 1000 classes. Once trained, the pre-trained DNN can be adopted for any image classification by changing the last layers of the DNN. In our study, pre-trained ResNet-18 [28] is adopted to classify the raw corneal images.

A few studies have utilized pre-trained DNNs to classify corneal images. The major drawbacks of these studies require complex preprocessing steps and segmented images, because the classification performance of the pre-trained networks is insufficient for raw corneal images. To handle this problem, we have proposed a novel technique to classify raw corneal images directly by combining the ResNet and the Genetic Algorithm (GA).

To compute the optimal vector of an optimization problem, meta-heuristic algorithms are used owing to global searching operations. The GA is a heuristic optimization technique applied to solve complex problems [29]. Compared to the classical optimization technique, the GA can be beneficial in optimizing the functions with many local minimums [29]. One of the most capable methods is the GA because of the evolutionary mechanizes including cross-over and mutation are modeled in the GA [30]. Therefore, exploration and exploitation processes of the GA is balanced for robust search. Moreover, The GA is well-known method in the global optimization algorithm [31]. In addition, the GA is reported as one of the most used methods for feature selection [32]. For this reason, the GA is used to select convenient image subsets from the ResNet layers.

Typically, the last three layers of the ResNet are changed, and the last fully connected layer and softmax layer weights are tuned to attain classification for the new data set. The classification performance depends on the features obtained at the output of the last feature extaction layer of ResNet before applied the proposed method. The output of the each layer can be employed to classify corneal ulcer. The each layers has been tested to find which one is the best by using GA. Recently, while the SVM as a classifier replaced with softmax for ResNet, it is reported that relatively higher accuracy is obtained in the literature [33,34,35,36]. To increase the classification performance of the proposed method, the SVM classifier is utilized. For further improvement, we select some image subsets from the layers mentioned above by using the GA, which eliminates the redundancy features in the image.

The main contribution of the paper can be summarized as follows:

1. An AI-based Corneal Ulcers detection method is proposed for Diagnosis support
2. The extracted features maps from each layer of ResNet is selected by the GA. Then selected feature maps are classified by the SVM in the proposed method.
3. The ResNet is used to extract features; therefore, the fine-tuning step is eliminated to save time and energy.
4. Instead of softmax, the SVM is used, which increases the algorithm’s performance.
5. The GA is utilized to select some image subsets from the layers of the ResNet to decrease the redundancy.
6. Major disadvantages of the DNN and pre-trained ResNet, including hyperparameter optimization, large data set requirements, time-consuming optimization process, etc., are eliminated for corneal image classification.

The rest of the paper is organized into three parts: Method, Results and Discussions, and Conclusion. The method section gives general information about the DNN, Transfer Learning, the ResNet, the GA, the SVM and the proposed algorithm. The detailed results with discussions are presented in the Section 3. The last section concludes the study.

## 2. Method

This section presents the fundamentals of deep convolutional neural network (CNN) based DNN, the used GA, the SVM and the fully structure of the proposed method.

### 2.1. Deep Convolutional Neural Network

A CNN based DNN consists of many convolutional and pooling layers and a fully connected layer as well. The parameters of convolutional and fully-connected layers are tuned during the training process. However, there is no parameter to be tuned in the pooling processing [37].

The convolutional layer (CL) has a bunch of neurons structured as an image with multiple depths. the CLs extract features, including edges, texture etc., from an input image [37]. Therefore, the CLs are accepted as tunable filters called the convolutional filters or convolutional kernels. In general, the size of a CL is n×m×d, where *n*, *m*, *d* are the input sizes. Each CL kernel computes convolving operation with the input image. In this computing, the dot product is realized between the filter entries and the input [38].

The pooling layer (PL) aims to downsample each convolved feature (CF). Thus, the needed computational cost is decreased thanks to dimensionality reduction. Consequently, the reduced size of CF is provided to control the overfitting problem [38]. A fully connected layer (FCL) maps the features from the last PL to the classes. the FCL is structured as conventional artificial neural networks [39].

All tunned parameters are fully connected to the subsequent layers in DNN models [38]. Indeed, because of computing cost, these fully-connected parameters are insufficient to classify problems, especially on images with many pixels. Hence, neurons consisting of a large number of weights cause rapid overfitting [39]. Some connections are dropouted in DNN models to overcome the overfitting problem. Moreover, pre-trained models, including AlexNet, ResNet, GoogleNet, DenseNet, etc., can be used to obtain a more robust DNN model. Using a pre-trained model for a different data set is defined as transfer learning.

#### 2.1.1. Transfer Learning

Utilization of a previously acquired ability in a novel task is defined as transfer learning [40]. Recently, many successful applications of transfer learning have been proposed in machine learning or data mining areas. Re-training for a new task with new data using trained DNN for a generic task has been accepted as transfer learning [41]. The computational cost is reduced, and the requirement of extensive data set is eliminated thanks to transfer learning. The most successful applications to perform transfer learning are based on the DNN models trained with ImageNet [27,42] in medical tasks [26,43].

#### 2.1.2. ResNet

When the DNNs begin converging an optimal local solution point, a degradation problem can arise in large-scale networks. As the layers of a DNN increase, the accuracy of the DNN becomes worse, saturates and degrades rapidly [44]. In the literature, this drawback is defined as a degradation problem, which causes the optimization process to stop. To overcome the degradation problem, the Residual neural network (ResNet) [44] has been proposed as a new DNN framework to classify a big data set called Imagenet [27]. The applied technique is simple, but the results are very efficient. Some connections and layers are jumped in the ResNet. Thus, the ResNet can solve the degradation problem.

**Figure 1:**
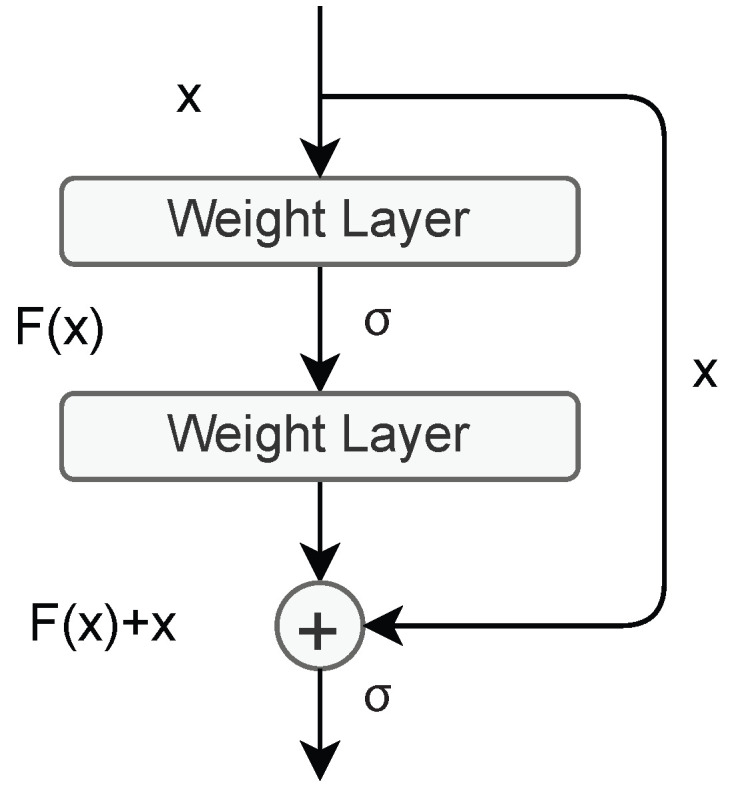
The Residual learning block.

The residual learning is shown in Figure 1. The residual learning can be implemented every few stacked layers. A residual building block is defined as:

(1) $$y=F\left(x,\left\{W_{i}\right\}\right)+x$$

Here, the input x and output y vectors are connected with the $F\left(x,\left\{W_{i}\right\}\right)$ function which is a residual mapping function without bias. In Figure 1, there are two layers. Their connections are computed as $F=W_{2}f\left(W_{1}x,\right)$, where *f* is presented as the ReLU function. The dimensions of x and *F* have to be equal in Equation (1) [44,45]. For this reason, the Equation (1) is reformed as below:

(2) $$y=F\left(x,\left\{W_{i}\right\}\right)+W_{s}x$$

where, $W_{s}$ is a square matrix to use a linear projection by the shortcut connections to match the dimensions.

In this study, the ResNet-18 architecture is used.

### 2.2. Genetic Algorithm

The genetic algorithm is a well-known heuristic search method for global optimization problems based on evolutionary strategy. The GA has been introduced by John Holland in the 1970s [46,47]. The GA is a stochastic search algorithm based on the mechanics of natural selection, crossover, and mutation operations. A chromosome is represented as a candidate solution in the GA. The GA begins a set of chromosomes, which is defined as population. The solutions are mined and developed over generations. At each generation, all chromosomes are evaluated to compute their fitness values. Each chromosome is selected as a partner according to fitness values. Selected chromosomes are as a parent, and then the parents produce a child as called offspring over crossover and mutation operations. The process of evolution is repeated until the end condition is satisfied or the maximum number of generations is reached [30,48]. The fundamental steps are presented in Algorithm 1.
**Algorithm 1** The fundamental steps of the genetic algorithm.1:**Initialization:**2:      Generate and evaluate randomly initial chromosomes.3:      Define the control parameters Crossover Rate (CR) and Mutation Rate (MR).4:**Repeat**5:      **Selection:**6:           Select chromosomes depending on the probability values according to selection strategy (best-fits).7:      **Crossover:**8:            Produce the new offsprings depends on crossover strategy over CR.9:      **Mutation:**10:           Apply the mutation to the new offspring as randomly over MR11:     Evaluate the new offsprings.12:     Replace least-fit population with new offspring.13:     **Keep the best offspring in the memory.**14:**Until** (Maximum generation number)

### 2.3. Support Vector Machine

The support vector machine has been proposed by Vapnik et al. [49,50]. The SVM is a machine learning method, which can be used for any classification, clustering, and regression problems [51]. The kernel function is utilized to map from input as high-dimensional features to output for the concerned problem in the SVM. The kernel function is called the support vector kernel. The success of SVM depends not only on the number of support vectors and weights but also on the kernel function [52]. Different kernels can be used, including the linear, Gaussian, quadratic, cubic and polynomial kernels concerning the nature of data sets. The linear kernel is used in the proposed method.

### 2.4. Proposed Method

Pre-trained models can be employed to classify almost all image types on the condition that a practical training process is performed on the networks with new images before. The most common pre-training models for transfer learning in medical image classification are the AlexNet, the GoogleNet, the DenseNet and the ResNet. The newest review paper, which deals with medical image classification using transfer learning has been published by Kim and et al [53]. The ResNet and Inception models are advised to employ the medical image classification problems by reviewing 425 transfer learning studies in the mentioned reviewed paper [53]. It should be noted that the ResNet model is more effective in extracting the features of medical images thanks to ability of overcome the degradation problem. In addition, while the computational complexity of the ResNet 18 model compared with the other version of ResNet is lower, the accuracy rates of ResNet models are almost the same [39,54,55,56]. In addition, the performance of the ResNet 18 model has been boosted thanks to the image selection to solve the corneal ulcer detection problem.

**Figure 2:**
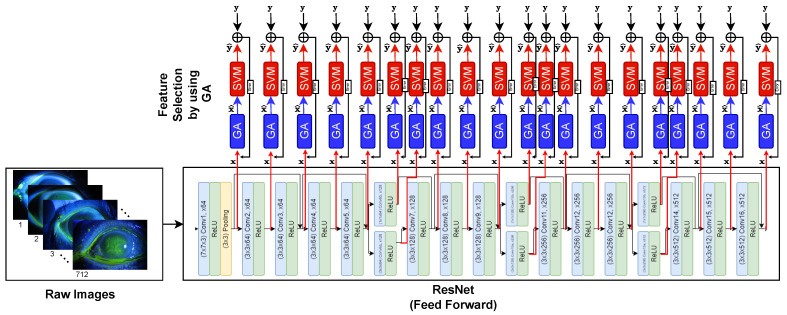
The framework of the proposed method. *x*: Features obtained on output of the related layer. $\hat{x}$: Selected Features obtained by GA. *y*: Actual label. $\hat{y}$: Predicted label.

**Figure 3:**
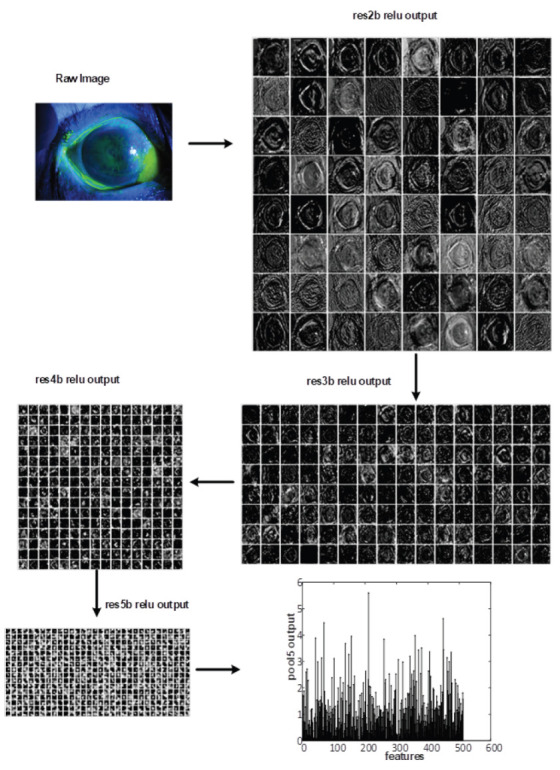
The features maps of selected layers on a raw image.

In this study, The GA, SVM, and ResNet are combined to detect the corneal ulcer from the raw images. The framework of the proposed method is illustrated in Figure 2. In the framework of the proposed method, the following steps are performed. First, the raw images are fed to the input of the ResNet. Next, the feature maps (x) are computed on the output of the handled layer of ResNet. Figure 3. presents an example of feature map extraction. Then, the effective feature maps ($\hat{x}$) are selected using the GA. After that, the averages of each selected feature map are calculated as pooling. Finally, the SVM is utilized to classify ($\hat{y}$) corneal ulcers from extracted and chosen features. Consequently, a more successful classifier method has been obtained to detect a corneal ulcer.

**Figure 4:**
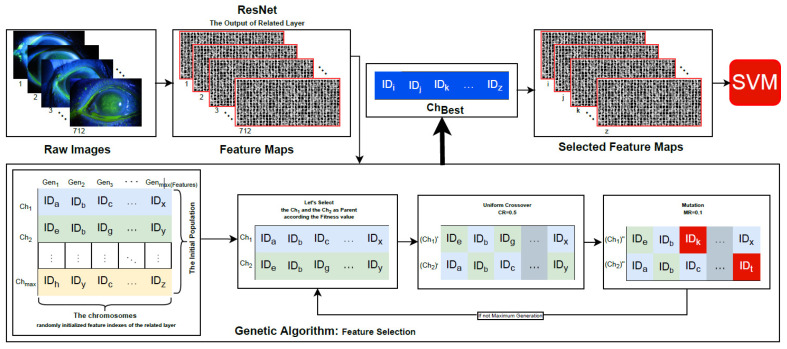
The feature selection framework of the proposed method.

The feature selection framework of the proposed method is shown in Figure 4. Since there are exactly 712 images in the dataset for corneal ulcers, the same number of feature maps are computed on each layer of ResNet. We aimed to select the most effective 192 feature maps in the proposed method. For this reason, the dimensionality of each chromosome in the GA is 192, where each gen is initialized randomly. The parents are selected according to fitness values. The fitness of each chromosome is equal to the accuracy of SVM over selected feature maps based on the related chromosome. The uniform crossover [57] is implemented in GA. A random value between [0,1] is generated for each gene in the uniform crossover. If the randomly generated value is less than CR = 0.5, the gene is assigned to the offspring ($Ch_{1}$). Otherwise, the gene is assigned to the offspring ($Ch_{2}$). The value of MR is advised to be between 0.05 and 0.2 for the exploitation [58]. Unfortunately, there is no numerical method to set the MR value. Each offspring is mutated on MR = 0.1 over trial-error method. A random value between [0,1] is generated for each gene in the mutation. If the randomly generated value is less than MR = 0.1, the randomly selected image index must be different from the genes of the chromosome and is assigned to the offspring. Otherwise, the gene is assigned to the other offspring. The best chromosome of each generation is stored. The selection parents, crossover and mutation operations are executed until maximum generation.

The control parameters of the proposed method is given in Table 1.

#### 2.4.1. Dataset

**Figure 5:**
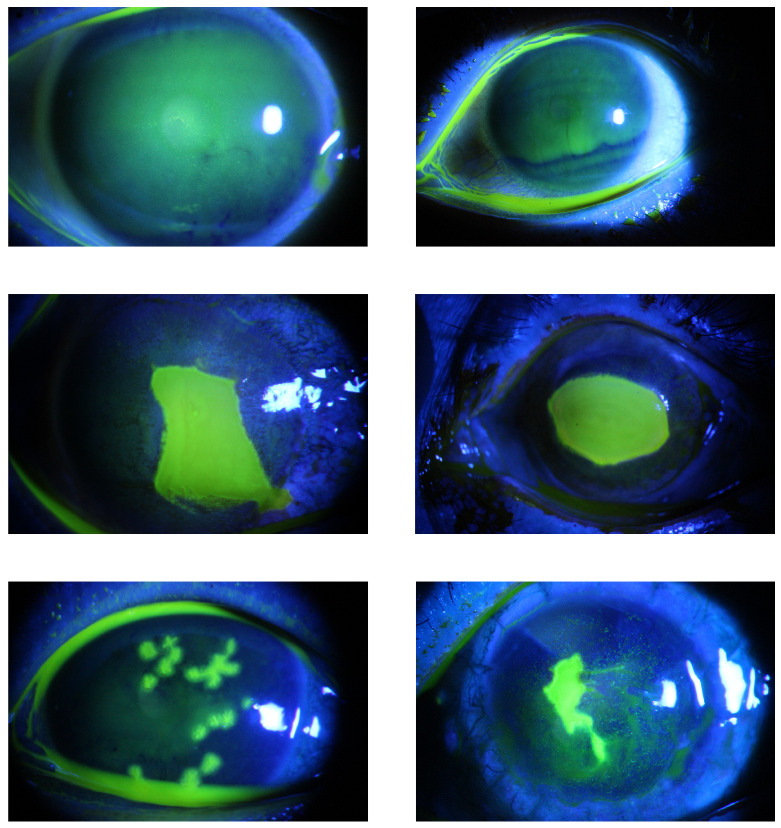
Corneal ulcers sample images from datasets.

A total of 712 fluorescent staining images capturing ocular surfaces have been collected from patients with varying degrees of corneal ulcers at the Zhongshan Ophthalmic Center at Sun Yat-sen University [5]. Slit-beam illumination with a maximum width of the white light source (30 mm) a blue excitation filter, a magnification of 10 or 16, and a diffusion lens at an oblique angle of 10 to 308 with the light source at the bottom and an automatic digital camera system have been utilized to adjust the aperture, exposure time, and shutter speed depending on the brightness of the examination room. The Images have been acquired using a Haag Streit BM 900 slit lamp microscope (Haag Streit AG, Bern, Switzerland) in conjunction with a Canon EOS 20D digital camera (Canon, Tokyo, Japan). The Images have been recorded in JPG format with 24-bit RGB color at 2592×1728 pixel resolution. Each image contains only one cornea, which is fully represented in the image and approximately centered in the field of view [5]. Some corneal ulcer sample images are presented in Figure 5 from the dataset.

#### 2.4.2. Evaluation Metrics

To evaluate the performance and effectiveness of the proposed method, the accuracy and computational time metrics have been used.

The accuracy is calculated by the following equation:

(3) $$Accuacy=\frac{TP+TN}{TP+TN+FP+FN}$$

where TP, TN, FP and FN are True Positive, True Negative, False positive and False Negative, respectively [32].

To compute the computational time analyzes, a metric was proposed in [59]. A reference program was presented in the technical report. The evaluation of the proposed method is the relationship to the computational time of the reference program. The computational time or complexity is calculated with the following equation.

(4) $$CT=\frac{\hat{T}1}{T0}$$

Here, while $\hat{T}1$ is the computing time of the proposed methods, T0 is the computing time of the reference program [59].

## 3. Results and Discussions

There are exactly 71 layers in the ResNet that are used in this study. These layers consist of the convolution layer, ReLu layer and pooling and normalization layers mentioned in the method section. By repeating these basic layers, the DNN gradually reveals the features in the data from the input to the output. Unlike traditional deep neural networks, ResNet has an extra normalization layer in each layer.

The ResNet consists of 10 blocks. Block-1 consists of input image, convolution, normalization, ReLu and pooling layers. Blok-2a consists of convolution, normalization, ReLu, convolution, normalization, element wise sum (Blok-1 output and Blok-2a last normalization layer output), ReLu (Blok-2a output), respectively. The next 7 blocks are structured similarly to Blok-2a. The last block has a fully connected layer as a classification layer.

**Figure 6:**
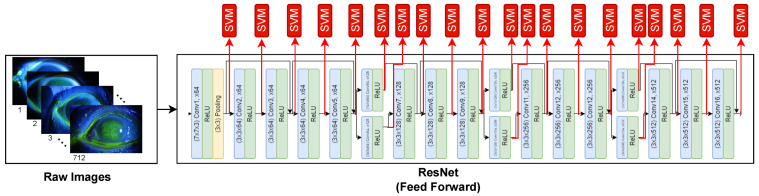
The analyzing process of general feature mapping over each convolutional layer.

In this study, which of the features obtained from ResNet’s 67 layers is more effective for classification performance has been examined. The examining process is shown in Figure 6. 3 of the ResNet layers have 112×112×64 (2,408,448) attributes, 15 of the ResNet layers are 56×56×64 (3,010,560), 16 of the ResNet layers are 28×28×128 (1,605,632), 16 of the ResNet layers are 14×14×256 (802,816), 16 of the ResNet layers are 7×7×512 (401,408), 1 of the ResNet layers are 1×1×512 (512). Approximately 8.2 million features have an indirect effect on the classification. In the classical approach, classification is made using 512 features in pool5, which is the last layer. However, with the attributes from pool5, the performance was only around 0.64. By applying the GA, success rates of around 0.85 were achieved with a correct layer selection strategy. This result clearly shows that ResNet, etc., structures have more than necessary attributes for such small-scale datasets.

In the experimental study, first of all, which layers affect the classification performance are examined. To accomplish this goal, a representation of each image from 67 layers is found. This representation is found by averaging each image. For example, the output of the $i_{th}$ layer can be represented by $a^{i}\times b^{i}\times w^{i}$ which contains $w^{i}$ images with $a^{i}\times b^{i}$ size converted to a $1\times w^{i}$ vector. Here $w^{i}=\left[w_{1}^{i}\;w_{2}^{i}\;...\;w_{m}^{i}\right]$ contains the mean of each image with sizes $a^{i}\times b^{i}$ where i=1,2,...,67. In this study, the data set is divided into two parts: 70% training and 30% testing.

**Figure 7:**
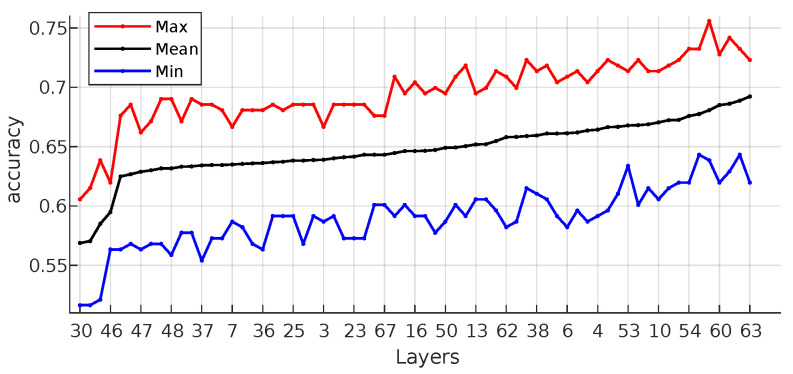
The Accuracy rates of each layers.

**Figure 8:**
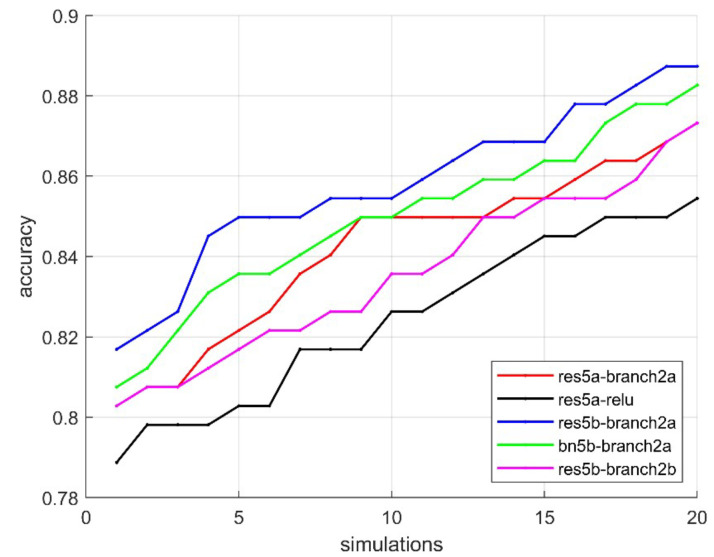
The accuracy rates of proposed method on each simulation.

Technically, each image output is expressed as an average number. Thus, the number of features has been effectively reduced. As a result, 18, 16, 16, 17 of the ResNet layers were reduced to 64, 128, 256, 512 features, respectively. The minimum, maximum, mean, median, and std values of 20 runs obtained from each layer are given in Table 2. According to this table, res5b_branch2b, res5a_relu, bn5b_branch2a, res5b_branch2a and res5a_branch2a layers have the highest classification performance. In addition, the success rates of the layers are given graphically in Figure 7. It can be seen in Figure 8, the success rates of res5b_branch2b, res5a_relu, bn5b_branch2a, res5b_branch2a and res5a_branch2a layers from each run are also given in detail. The summary of Figure 8 can be seen in Table 3. All of these layers are generally located at the end of the ResNet. It has been observed that the classification performance increases as the network structure approaches the end. It should be noted that the result of the pool5 layer handled by the classical approach fell behind many layers with an accuracy value of 0.64.

**Figure 9:**
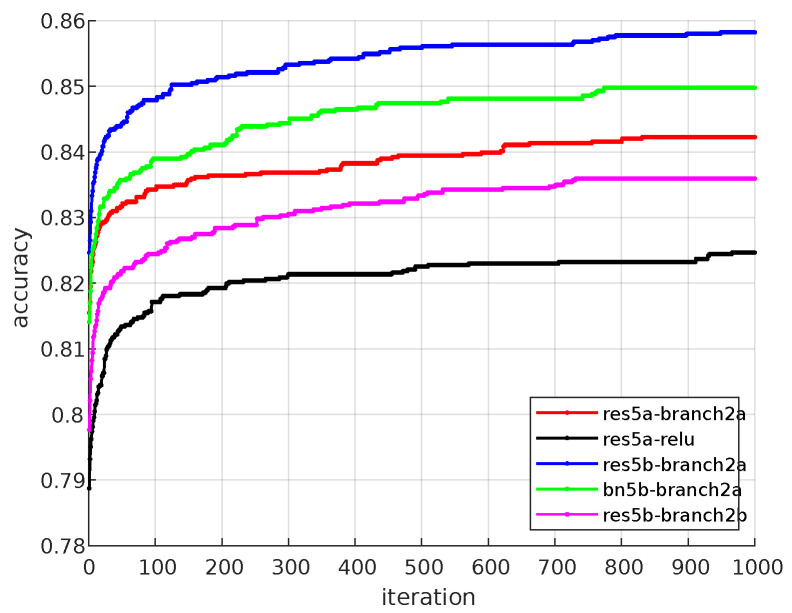
Average convergence graphs of the best five layers.

As a result of this preprocessing, images obtained from res5b_branch2b, res5a_relu, bn5b_branch2a, res5b_branch2a and res5a_branch2a layers are studied in more detail. From the output of these layers, 512 images of 7 sizes are obtained. In the study, it was emphasized which of these 512 images could be more effective in classification. First, a certain group of images was selected by trial and error and sent to the SVM classifier. After a certain improvement in the results obtained, the images were selected more systematically with the help of GA. While applying the GA, the population size was selected as 40 and the chromosome number (image number) as 192. The mutation rate was determined as 0.1 and the number of iterations was chosen as 1000. Since there is no systematic method for selecting these parameters, the parameters were chosen by trial and error. In addition, convergence graphs of res5b_branch2b, res5a_relu, bn5b_branch2a, res5b_branch2a and res5a_branch2a layers are given in Figure 9 where considerable improvement can be observed until 400 iterations for all layers.

Although such a process is extremely time-consuming and exhausting, the classification performances obtained are extremely high. Extremely high success rates are obtained by increasing the average performance from 0.64 to 0.67 with layer selection and 0.86 with image selection. It can be seen from Table 4, huge performance increases were observed for res5b_branch2b, res5a_relu, bn5b_branch2a, res5b_branch2a and res5a_branch2a layers. The gains of proposed method are between 19.73 and 25.28. These results are a clear proof of how much unnecessary detail the deep neural network may contain.

The results obtained from res5b_branch2b, res5a_relu, bn5b_branch2a, res5b_branch2a and res5a_branch2a layers are also compared with each other statistically. The comparison is shown in Table 3 over the basic statistical values. The Wilcoxon test is a non-parametric statistical test based on the mean accuracy for checking statistically difference of two methods [32]. For this reason, the Wilcoxon signed-rank test is utilized to expose the success of selected maps from layers. The results of Wilcoxon signed-rank test is reported in Table 5. According to the results shown in this table, there is no significant difference between res5b_branch2b and res5a_branch2a (*p*-value > 0.05), but there is a statistically significant difference in all other combinations.

The article [53] has been published by the owners of the dataset used in this study. To compare the results of the proposed method with the results of other methods in which used the same dataset in the literature, the total 40 papers cited to the article [53] were initially retrieved from Web of Science (11), PubMed (7) and Google Scholar (22) databases. 25 of them were ignored because of duplicate papers. The remaining 15 papers were unique and were assessed for comparison. The segmentation (5) and the medical (3), a total of 8 studies were excluded due to being not focused on the classification. In the remaining 5 studies, corneal ulcer types, which are point-like corneal, point-flaky mixed corneal and flaky corneal ulcers, has been aimed to classify without detecting corneal ulcer using the transfer learning [60,61,62,63,64]. The details of the mentioned publications [60,61,62,63,64] are presented in Table 6. The binary classification s being corneal ulcer or not being has been aimed a in our study. In the last remaining studies, the images have been masked before feeding the their proposed methods. Consequently, there is not any study present in the literature for a fair comparison, to the best of our knowledge.

Computational time (CT) is an important parameter to evaluate an algorithm’s efficiency. To calculate the CT of the proposed method, the recommended method for a meta-heuristic approach in the technical report [59] is utilized. The control parameters of the proposed method are used as presented in Table 1 for computing feature maps selection (FMS) and classification over selected feature maps (SFM). The simulations have been performed on a PC i3-7130U 2.7 GHz, 20 GB RAM. The calculated CTs of the proposed method is given in Table 7. When this table is analyzed, it is seen that the CTs of the FMS process on each layer are high computing values. Although, the CTs of the classification over SFMs on each layer are acceptable computing values. However, these facts are negligible thanks to the gain (nearly %25) in the classification performance of the proposed methods.

## 4. Conclusions

The results presented in this study reveal how good results can be obtained when the images formed in the inner layers of the ResNet. The study has provided to reveal and analyze the disadvantages that occur when a network structure with many layers such as the ResNet is used as a feature extractor. This study consists of the main frameworks including the ResNet, GA and SVM. In future studies, it may be possible to obtain higher performances by trying different versions of these structures. The most important problem encountered in this study is that selecting an image from a structure such as the ResNet with the GA is a very time-consuming process. To solve this problem, the population size can be reduced. However, in this case, the classification performance decreases. Optimum population size is extremely critical. Our method has superior performance over the conventional Resnet18; however, to generalize the proposed methods, we need experimental extension setups, including large-scale pre-trained DNNs and large-scale data sets. However, the DNN with the GA needs too much time to run in a large-scale network and large-scale data set. Therefore, the proposed method is suitable for small or medium-scale data sets with small-scale DNN. Moreover, successes of recently proposed attention module based residual network is remarkable for AI problems. The proposed strategy could be adopted to neural attention network to improve the achievement.

## Figures and Tables

**Table 1:** The parameters of the proposed method.

	Parameter Name	Parameter Value
Feature Extraction (Deep Model)	Architecture	ResNet-18
	Fine Tunning	No
	Input	Raw Images
	Output	Feature Maps
Feature Selection (GA)	Population Size	40
	CR	0.5
	MR	0.1
	Max Gen	1000
Classifier (SVM)	SVM-Kernel	Linear Kernel
Selected Feature Maps	Input size	712
	Output size	192

**Table 2:** Accuracy rates results of ResNet based on each layer with classification (Sorted according to mean accuracy rates).

Layer	Layer Name	Mean	Max	Min	Median	Layer	Layer Name	Mean	Max	Min	Median
**63**	**res5b_branch2b**	**0.6923**	0.723	0.6197	0.7042	**16**	bn2b_branch2b	0.6462	0.7042	0.5915	0.6408
**59**	**res5a_relu**	**0.6887**	0.7324	0.6432	0.6925	**18**	res2b_relu	0.6446	0.7089	0.5915	0.6455
**61**	**bn5b_branch2a**	**0.6862**	0.7418	0.6291	0.6831	**66**	res5b_relu	0.6432	0.6761	0.6009	0.6502
**60**	**res5b_branch2a**	**0.685**	0.7277	0.6197	0.6831	**67**	pool5	0.6432	0.6761	0.6009	0.6502
**51**	**res5a_branch2a**	**0.6808**	0.7559	0.6385	0.6761	**34**	res3b_relu	0.6432	0.6854	0.5728	0.6502
**58**	res5a	0.6775	0.7324	0.6432	0.6737	**23**	res3a_branch2a_relu	0.6415	0.6854	0.5728	0.6408
**54**	bn5a_branch2a	0.6758	0.7324	0.6197	0.6784	**32**	bn3b_branch2b	0.6411	0.6854	0.5728	0.6479
**57**	bn5a_branch2b	0.6725	0.723	0.6197	0.6761	**24**	res3a_branch2b	0.6401	0.6854	0.5915	0.6455
**12**	res2b_branch2a	0.6723	0.7183	0.615	0.6784	**3**	conv1_relu	0.639	0.6667	0.5869	0.6479
**10**	res2a	0.6704	0.7136	0.6056	0.6761	**8**	res2a_branch2b	0.6387	0.6854	0.5915	0.6432
**11**	res2a_relu	0.6688	0.7136	0.615	0.6737	**25**	bn3a_branch2b	0.6383	0.6854	0.5915	0.6432
**42**	res4a	0.6681	0.723	0.6009	0.6714	**26**	res3a	0.6383	0.6854	0.5681	0.6432
**53**	bn5a_branch1	0.6678	0.7136	0.6338	0.662	**20**	res3a_branch1	0.6373	0.6808	0.5915	0.6385
**40**	res4a_branch2b	0.6667	0.7183	0.6103	0.669	**9**	bn2a_branch2b	0.6369	0.6854	0.5915	0.6455
**41**	bn4a_branch2b	0.6664	0.723	0.5962	0.669	**36**	res4a_branch1	0.6362	0.6808	0.5634	0.6432
**4**	pool1	0.6643	0.7136	0.5915	0.6714	**39**	res4a_branch2a_relu	0.6359	0.6808	0.5681	0.6385
**5**	res2a_branch2a	0.6636	0.7042	0.5869	0.6667	**28**	res3b_branch2a	0.6354	0.6808	0.5822	0.6455
**49**	res4b	0.662	0.7136	0.5962	0.6714	**7**	res2a_branch2a_relu	0.635	0.6667	0.5869	0.6455
**6**	bn2a_branch2a	0.6613	0.7089	0.5822	0.6643	**55**	res5a_branch2a_relu	0.6345	0.6808	0.5728	0.6385
**56**	res5a_branch2b	0.661	0.7042	0.5915	0.6761	**31**	res3b_branch2b	0.6345	0.6854	0.5728	0.6338
**35**	res4a_branch2a	0.661	0.7183	0.6056	0.6549	**37**	bn4a_branch1	0.6343	0.6854	0.554	0.6362
**38**	bn4a_branch2a	0.6594	0.7136	0.6103	0.6596	**27**	res3a_relu	0.6333	0.6901	0.5775	0.6432
**44**	res4b_branch2a	0.6589	0.723	0.615	0.662	**21**	bn3a_branch1	0.6331	0.6714	0.5775	0.6432
**19**	res3a_branch2a	0.6582	0.6995	0.5869	0.6667	**14**	res2b_branch2a_relu	0.6317	0.6901	0.5681	0.6244
**62**	res5b_branch2a_relu	0.658	0.7089	0.5822	0.6667	**48**	bn4b_branch2b	0.6317	0.6901	0.5587	0.6362
**17**	res2b	0.6547	0.7136	0.5962	0.6526	**29**	bn3b_branch2a	0.63	0.6714	0.5681	0.6362
**45**	bn4b_branch2a	0.6521	0.6995	0.6056	0.6573	**47**	res4b_branch2b	0.6289	0.662	0.5634	0.6338
**13**	bn2b_branch2a	0.6519	0.6948	0.6056	0.662	**65**	res5b	0.6268	0.6854	0.5681	0.6197
**52**	res5a_branch1	0.6505	0.7183	0.5915	0.6549	**64**	bn5b_branch2b	0.6249	0.6761	0.5634	0.615
**43**	res4a_relu	0.6493	0.7089	0.6009	0.6549	**46**	res4b_branch2a_relu	0.5948	0.6197	0.5634	0.5962
**50**	res4b_relu	0.6491	0.6948	0.5869	0.6596	**1**	conv1	0.585	0.6385	0.5211	0.5892
**33**	res3b	0.6472	0.6995	0.5775	0.6502	**2**	bn_conv1	0.5704	0.615	0.5164	0.561
**22**	bn3a_branch2a	0.6465	0.6948	0.5915	0.6479	**30**	res3b_branch2a_relu	0.5688	0.6056	0.5164	0.5681
**15**	res2b_branch2b	0.6462	0.6948	0.6009	0.6385						

The obtained five highest accuracy layers of the ResNet are bolded.

**Table 3:** The descriptive statistical on accuracy rates of the proposed method over the best five layer of the ResNet.

Layer	Mean	Max	Min	Median	Std
res5b_branch2a	0.8582	0.8873	0.8169	0.8568	0.0204
bn5b_branch2a	0.8498	0.8826	0.8075	0.8521	0.0214
res5a_branch2a	0.8423	0.8732	0.8028	0.8498	0.0215
res5b_branch2b	0.8359	0.8732	0.8028	0.8357	0.0214
res5a_relu	0.8246	0.8545	0.7887	0.8263	0.0212

**Table 4:** The gain of the proposed method.

Layer	Proposed Method Mean AR	Feed Forward Mean AR	The Difference	Gain %
res5b_branch2a	0.8582	0.685	0.1732	25.28
bn5b_branch2a	0.8498	0.6862	0.1636	23.84
res5a_branch2a	0.8423	0.6808	0.1615	23.72
res5b_branch2b	0.8359	0.6923	0.1436	20.74
res5a_relu	0.8246	0.6887	0.1359	19.73

**Table 5:** The results of Wilcoxon statistic test on accurate rates of the best five layer over 20 independent runs.

Layers	res5a_branch2a	res5a_relu	res5b_branch2a	bn5b_branch2a	res5b_branch2b
res5a_branch2a	1	0.0004	0.0002	0.0045	0.0555
res5a_relu	0.0004	1	0.0001	0.0001	0.0025
res5b_branch2a	0.0002	0.0001	1	0.0029	0.0003
bn5b_branch2a	0.0045	0.0001	0.0029	1	0.0014
res5b_branch2b	0.0555	0.0025	0.0003	0.0014	1

**Table 6:** Performances of the other pre-trained networks.

Publication	Accuray (%)	Training Method	Details of Method
Daoud and et al. [60] (2022)	76.3	N/A	Vertex AI based method was proposed. However, there was no information about the architecture of proposed method and training procedures.
Alquran and et al. [61] (2022)	65.8	70% training and 30% testting	1. ResNet based method was proposed. 2. The dataset was augmented and manuel feature extraction process is implemented by expert. 3. Before classification, dimensionality reduction methods including ECFS and PCA was utilized.
Lv and et al. [62] (2022)	N/A	5-fold cross-validation	1. MIF-Net based on DenseNet method was proposed. 2. The accuracy scores were not presented. However, recall and F1 scores were given as 87.07 and 86.82, respectively
Gross and et al. [63] (2021)	66.4	80% training and 20% testting	1. The CNN based method was proposed. 2. The corneal ulcer was labeled as early and advanced stages for binary classification
Cinar and et al. [64] (2021)	80.42	80% training and 20% testting	1. AlexNet based method was proposed. 2. The dataset was augmented process is implemented.

**Table 7:** The computational times of the proposed method.

Layers	CTs of FS			CTs of Classification over SFMs		
	Mean	Std	C	Mean	Std	C
res5b_branch2a	$4.68\times 10^{3}$	$1.84\times 10^{2}$	$1.46\times 10^{4}$	$6.80\times 10^{-2}$	$6.11\times 10^{-3}$	$2.12\times 10^{-1}$
bn5b_branch2a	$4.42\times 10^{3}$	$1.26\times 10^{2}$	$1.38\times 10^{4}$	$6.27\times 10^{-2}$	$6.69\times 10^{-3}$	$1.95\times 10^{-1}$
res5a_branch2a	$4.46\times 10^{3}$	$2.90\times 10^{2}$	$1.39\times 10^{4}$	$8.23\times 10^{-2}$	$5.44\times 10^{-2}$	$2.56\times 10^{-1}$
res5b_branch2b	$4.25\times 10^{3}$	$1.22\times 10^{2}$	$1.32\times 10^{4}$	$6.71\times 10^{-2}$	$1.82\times 10^{-2}$	$2.09\times 10^{-1}$
res5a_relu	$4.36\times 10^{3}$	$3.12\times 10^{2}$	$1.36\times 10^{4}$	$6.28\times 10^{-2}$	$9.09\times 10^{-3}$	$1.95\times 10^{-1}$
Avg. of the rows	$4.44\times 10^{3}$	$2.07\times 10^{2}$	$1.38\times 10^{4}$	$6.86\times 10^{-2}$	$1.89\times 10^{-2}$	$2.13\times 10^{-1}$

## Data Availability

The dataset is available in below URL as mentioned by the original authors in [5]. https://github.com/CRazorback/The-SUSTech-SYSU-dataset-for-automatically-segmenting-and-classifying-corneal-ulcers (accessed on 17 April 2022).
